# Role of *Sinorhizobium meliloti* and *Escherichia coli* Long-Chain Acyl-CoA Synthetase FadD in Long-Term Survival

**DOI:** 10.3390/microorganisms8040470

**Published:** 2020-03-26

**Authors:** Ángel de la Cruz Pech-Canul, Geovanny Rivera-Hernández, Joaquina Nogales, Otto Geiger, María J. Soto, Isabel M. López-Lara

**Affiliations:** 1 Programa de Ecología Genómica, Centro de Ciencias Genómicas, Universidad Nacional Autónoma de México. Cuernavaca, Morelos, C.P. 62210, Mexico; angelpechcanul@gmail.com (Á.d.l.C.P.-C.); chambolom@gmail.com (G.R.-H.); otto@ccg.unam.mx (O.G.); 2 Departamento de Microbiología del Suelo y Sistemas Simbióticos. Estación Experimental del Zaidín, CSIC, 18008 Granada, Spain; jnogales@noble.org (J.N.); mariajose.soto@eez.csic.es (M.J.S.)

**Keywords:** long-chain acyl-CoA synthetase, free fatty acids, *Sinorhizobium (Ensifer)*, surface motility, survival, malonyl-CoA synthetase

## Abstract

FadD is an acyl-coenzyme A (CoA) synthetase specific for long-chain fatty acids (LCFA). Strains mutated in *fadD* cannot produce acyl-CoA and thus cannot grow on exogenous LCFA as the sole carbon source. Mutants in the *fadD* (*smc02162*) of *Sinorhizobium meliloti* are unable to grow on oleate as the sole carbon source and present an increased surface motility and accumulation of free fatty acids at the entry of the stationary phase of growth. In this study, we found that constitutive expression of the closest FadD homologues of *S. meliloti*, encoded by *sma0150* and *smb20650*, could not revert any of the mutant phenotypes. In contrast, the expression of *Escherichia coli fadD* could restore the same functions as *S. meliloti fadD*. Previously, we demonstrated that FadD is required for the degradation of endogenous fatty acids released from membrane lipids. Here, we show that absence of a functional *fadD* provokes a significant loss of viability in cultures of *E. coli* and of *S. meliloti* in the stationary phase, demonstrating a crucial role of fatty acid degradation in survival capacity.

## 1. Introduction

Fatty acid metabolism has been studied mainly in the model organism *Escherichia coli* that can use long-chain fatty acids (LCFA) as sole carbon and energy source. The fatty acid degradation (Fad) pathway is responsible for the transportation, activation, and β-oxidation of LCFA (>10 carbons). These LCFA are transported into the cell by the outer membrane protein FadL and subsequently converted to their coenzyme A (CoA) thioesters by the enzyme acyl-CoA synthetase, encoded by *fadD* [1]. The degradation of acyl-CoAs proceeds via an inducible set of enzymes that catalyse the β-oxidative cleavage of the acyl-CoA into acetyl-CoAs. The first step in the β-oxidation cycle involves the conversion of acyl-CoA to enoyl-CoA via FadE. The remaining steps of hydration, oxidation and thiolytic cleavage in fatty acid degradation are performed by a tetrameric complex consisting of two copies each of FadA and FadB. The strains mutated in *fadD* cannot produce acyl-CoA and thus cannot grow on exogenous fatty acids [1].

Soto et al. [2] identified the *fadD* gene in the symbiotic nitrogen-fixing bacterium *Sinorhizobium (Ensifer) meliloti* GR4. Interestingly, in addition to being unable to grow on oleic acid as the sole carbon source, the mutation of *fadD* in *S. melitoti* GR4 resulted in multicellular swarming behaviour and defects in the establishment of symbiosis with alfalfa host plants. In agreement with these results, an increase of expression of motility genes and a decrease in nodulation gene expression were found in the *fadD* mutant, suggesting a FadD-dependent regulation mechanism [2]. In a follow-up investigation carried out to determine differences in the lipidic composition between *fadD* mutants and wild type, we found that strains of *S. meliloti* and of *E. coli* lacking functional FadD accumulated significant amounts of free fatty acids upon entry to the stationary phase of growth. We showed that fatty acids, accumulated in the *fadD* mutant, were derived from complex membrane lipids without the occurrence of cell lysis. Furthermore, the expression analysis of cultures showed the upregulation of genes involved in fatty acid degradation in *S. meliloti* wild type with respect to its *fadD* mutant strain [3], indicating that fatty acids released from membrane lipids are degraded by β-oxidation in the stationary phase of growth. However, the accumulation of free fatty acids was not responsible for the swarming phenotype observed in a *S. meliloti fadD*-mutant. Instead, the *fadD*-associated swarming phenotype was due to increased formation of the volatile 2-tridecanone [4].

Whereas *E. coli* K12 contains a unique *fadD* gene, *S. meliloti* Rm1021 contains several Open Reading Frames (ORFs) with homology to long-chain fatty acyl-CoA ligases [2]. A deeper analysis of the *S. meliloti* Rm1021 genome identified that, besides FadD, a total of nine ORFs with homology to acyl-CoA synthetases are present (Table S1, Figure S1). Interestingly, each one of the two closest FadD homologues are located in different replicons of *S. meliloti* Rm1021. The chromosomally encoded FadD of *S. meliloti* (SMc02162) shows a 55% identity to *E. coli* FadD, while its closest *S. meliloti* homologues SMb20650 (encoded in megaplasmid pSymB) and SMa0150 (encoded in megaplasmid pSymA), show an identity of about 30% to both the *E. coli* and the chromosome-encoded *S. meliloti* FadD (Table S1). Both SMb20650 and SMa0150 contain an AMP-binding motif as well as sequences that partially resemble the fatty acyl-CoA synthetase [FACS] signature motif common to all fatty acyl-CoA synthetases [2].

SMb20650 is a predicted long-chain fatty acyl-CoA ligase and its gene is cotranscribed with the gene coding for acyl carrier protein (ACP) SMb20651. Based on this, we hypothesized that SMb20650 could be involved in the acylation of SMb20651. The production of holo-SMb20651 in *E. coli* was achieved by co-expressing SMb20651 together with the phosphopantetheinyl transferase AcpS of *S. meliloti*. Additional expression of SMb20650 in the holo-SMb20651-forming *E. coli* strain, led to the in vivo formation of acylated SMb20651 [5]. SMa0150 shows a 75% identity to *Rhizobium leguminosarum* bv *trifolii* malonyl-CoA synthetase (MatB), a 67% identity to *Bradyrhizobium japonicum* MatB, and a 39% identity to *Streptomyces coelicolor* MatB. The activity as malonyl-CoA synthetase has been demonstrated in vitro for the latter three enzymes [6,7,8]. Importantly, the conserved motif ERYGMTE found in prokaryotic as well as in mammalian and plant malonyl-CoA synthetases [9,10] is present in SMa0150 (Figure S2). In *S. meliloti,* the gene coding for SMa0150 (MatB) is part of the operon *matPQMAB* that is induced by malonate, and a null mutant in *matB* is unable to grow on minimal medium containing malonate as the sole carbon source [11]. On the other hand, it was shown that the MatB of *R. leguminosarum* as well as the MatB of *S. coelicolor* have a broad substrate specificity [8,12].

In *Pseudomonas aeruginosa*, a total of six FadD homologues were functionally complementing an *E. coli fadD* mutant for its ability to grow in media containing fatty acids as the sole carbon source [13,14]. In the present study, we investigated whether the constitutive expression of either of the two closest homologues to *S. meliloti* FadD, SMb20650, or SMa0150, can complement the phenotypes exhibited by the *fadD* mutants of *S. meliloti* or *E. coli*. Given the pleiotropic phenotype of an *S. meliloti fadD* mutant and the suggested function of *S. meliloti* FadD as a regulator of expression [2], we also examined whether *E. coli* FadD reverts different phenotypes observed in a *S. meliloti fadD* mutant. Importantly, our studies reveal a crucial role of fatty acid degradation for survival in the stationary phase.

## 2. Materials and Methods

### 2.1. Bacterial Strains, Plasmids, and Growth Conditions

Bacterial strains and plasmids used in this work are listed in Table 1. *E. coli* strains were grown at 30 °C either in Luria-Bertani (LB) broth or in M9 minimal medium [15]. *Sinorhizobium meliloti* strains were grown at 30 °C either in complex tryptone yeast (TY) broth supplemented with 4.5 mM CaCl_2_ [16], in Robertsen minimal medium (MM) containing glutamate (6.5 mM), mannitol (55 mM), mineral salts (1.3 mM K_2_HPO_4_, 2.2 mM KH_2_PO_4_, 0.6 mM MgSO_4_, 0.34 mM CaCl_2_, 22 µM FeCl_3_, 0.86 mM NaCl), vitamins (biotin (0.2 mg/L), and calcium pantothenate (0.1 mg/L)) [17], or in Sherwood MM with succinate (8.3 mM), replacing mannitol as the carbon source [18]. To test the ability to use oleate as sole carbon source, 5 mM oleate (Sigma) was used in defined media with the addition of 5 mg/mL Brij 58 and for the case of Robertsen MM, 2 mM NH_4_Cl was used as the nitrogen source instead of glutamate. Antibiotics were added, when required, to the following final concentrations (μg/mL): carbenicillin, 100 and cloramphenicol, 20 for *E. coli*, or neomycin, 200 and tetracycline, 8 for *S. meliloti*. The mutation of the *fadD* gene in *E. coli* BL21 (DE3) was transduced from the *fadD* mutant JW1794-1 of the Keio Collection [19] by P1_vir_ transduction [20] selecting for kanamycin resistant colonies. Correct transfer of the *fadD* mutation in strain BfadD1 was corroborated by the absence of growth after plating on M9 MM agar plates containing 5 mM sodium oleate as unique carbon source.

### 2.2. DNA Manipulations

Recombinant DNA techniques were carried out using standard procedures [15]. Restriction sites introduced with oligonucleotides primers are underlined. The gene *sma0150* was amplified by PCR using specific primers (5′-ACCTTATCCATGGGCAACCATCTGTTCGACG-3′ and 5′-AAAGGATCCCTACACACGCGCTTCGGCTC-3′) and after digestion of the resulting fragment with *Nco*I and *Bam*HI it was cloned into pET16b, yielding plasmid pECH1. The gene *smc02162* was amplified by PCR using specific primers (5′-AGGAATCATATGGCGGAAGCAAGCACGC-3′ and 5′-CCCAAGCTTCTATCCGCGCAGGTCCTTG-3′) and after digestion of the resulting fragment with *Nde*I and *Hind*III it was cloned into pET17b, yielding plasmid pECH6. The *E. coli fadD* gene was amplified by PCR using specific oligonucleotides (5′-AAATTCACCATGGTAACGGCATGTATATCATTTG-3′ and 5′-ACAGGATCCTCAGGCTTTATTGTCCACTTTGC-3′) and after digestion of the resulting fragment with *Nco*I and *Bam*HI it was cloned into pET16b, yielding plasmid pECH8. Amplified DNA fragments were commercially sequenced by Eurofins Medigenomix (Martinsried, Germany) to confirm PCR fidelity. The gene *smb20650* was obtained by *Nde*I/*Eco*RI digestion from pAL55 [5] and subcloned into pET17b, yielding pECH7. The pET constructions were linearized and cloned into pRK404 previously digested with *Bam*HI or *Hind*III. The pRK404 derivatives were mobilized into *S. meliloti* QS77 by biparental mating using the *E. coli* S17-1 donor strain as previously described [22].

### 2.3. In vivo Labeling of S. meliloti and E. coli with ^14^C-Acetate and Analysis of Lipid Extracts by Thin-Layer Chromatography (TLC) 

The lipid composition of the different *S. meliloti* and *E. coli* strains was determined following labelling with [1-^14^C]-acetate as previously described [3]. Lipids from cell pellets were extracted according to the method of Bligh and Dyer [29] and lipids from spent media supernatants were extracted with equal volumes of acidified ethyl acetate (0.1 mL glacial acetic acid per litre of ethyl acetate). Lipids obtained were analysed by one-dimensional thin-layer chromatography (TLC) using high-performance TLC silica gel 60 plates (Merck) and mobile-phase ethyl acetate-hexane-acetic acid (60:40:5 (*v/v/v*)). Radioactivity was detected using a Storm 820 PhosphorImager (Amersham Biosciences). Image analysis and signal quantification were carried out using ImageQuant TL (Amersham Biosciences). *E. coli* BL21 (DE3)-derived strains were grown in M9 MM, and protein expression was induced by the addition of 0.1 mM isopropyl-β-D-thiogalactopyranoside (IPTG) during the mid-exponential phase of bacterial growth (OD_620nm_ = 0.4). The cultures were collected 21 h after induction with IPTG. For labelling experiments, *S. meliloti* strains were grown on Robertsen MM. Cultures were labelled at OD_620nm_ = 0.1 and collected after 66 h of growth. For each strain, labelling experiments were repeated 3 times and representative TLCs are shown.

### 2.4. Surface Motility Assays

The ability of *S. meliloti* strains to spread over surfaces was assayed essentially as previously described [30]. Briefly, *S. meliloti* was grown at 30 °C in TY broth to the late exponential phase. Cells were sedimented, washed twice in Robertsen MM, and resuspended in 1/10 of the initial volume. Aliquots of 2 µL of this cell suspension were dispensed onto the surface of plates with 20 mL of semisolid Robertsen MM containing 0.6% Noble Agar Difco (BD) and allowed to dry for 10 min. The plates were incubated at 30 °C for 24 h. Pictures and measurements of the migration zones (determined as the colony diameter in millimetres) were taken two days later to allow for the accumulation of exopolysaccharides and a better visualization of the colonies.

### 2.5. Cell Viable Counts of S. meliloti and E. coli

Cell viability from liquid cultures of *S. meliloti* and *E. coli* strains was followed by determining colony forming units (CFU) at distinct time points [31]. Colonies growing on plates were counted after 18 h of incubation for *E. coli* or 48 h for *S. meliloti*. The volume of *S. meliloti* liquid cultures in the long-term cultivation experiment carried out in Robertsen MM was kept constant by adding sterile distilled water. The experiments were independently performed three times with three replicates for each dilution. The cell viability was expressed as CFU/mL of culture.

## 3. Results

### 3.1. Growth of Sinorhizobium meliloti and Escherichia coli fadD Mutants on Oleate Cannot be Complemented by sma0150 or smb20650

As expected, mutation of the gene coding for long-chain fatty acyl-CoA synthetase in *S. meliloti* (*smc02162* = *fadD*) abolished the capacity to grow on oleate as sole carbon source [2]. Furthermore, this mutation leads to a swarming phenotype [2] and to the accumulation of endogenous free fatty acids [3]. In the present work, we investigated if all these phenotypes could be restored by heterologous complementation with *E. coli fadD* or by constitutive expression of *sma0150* or *smb20650*, encoding the two closest FadD homologues of *S. meliloti*.

**Figure 1:**
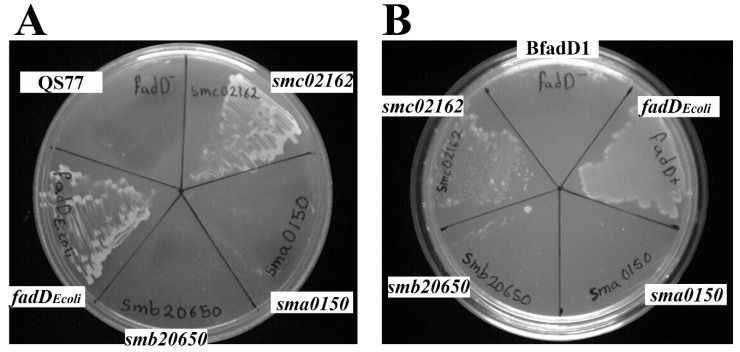
Expression of *smb20650* or *sma0150* in *S. meliloti* or *E. coli fadD* mutants does not restore growth on oleate. (**A**) The growth of *S. meliloti fadD* mutant QS77 carrying the empty vector pNG28 (QS77), pRCanul2 (*smc02162*), pRCanul1 (*sma0150*), pRCanul3 (*smb20650*), or pRCanul4 (*fadD_Ecoli_*) on Robertsen minimal medium (MM) with 5 mM oleate as a sole carbon source and 5 mg/mL Brij 58. (**B**) The growth of *E. coli fadD* mutant BfadD1 pLysS carrying pET17b (BfadD1), pECH8 (*fadD_Ecoli_*), pECH1 (*sma0150*), pECH7 (*smb20650*), or pECH6 (*smc02162*) on M9 MM containing 5 mM oleate as a sole carbon source, 5 mg/mL Brij 58, and 0.1 mM isopropyl-β-D-thiogalactopyranoside (IPTG). *S. meliloti* QS77 pNG28 and *E. coli* BfadD1 pLysS pET17b were used as controls.

Previously, it was demonstrated that plasmid pBBRD4 carrying *S. meliloti* GR4 *fadD* under its own promoter was able to restore growth on oleate in an *E. coli fadD* mutant [2]. In this study, we have cloned the DNAs coding for ORFs *smc02162* (*fadD*), *sma0150 (matB)*, *smb20650*, and *E. coli fadD* in pET vectors (see Table 1) and afterwards, the different expression vectors have been recloned into the broad host range vector pRK404. As a control, a cointegration of vectors pET17b and pRK404 (pNG28) was used. Growth on oleate of strain QS77 can be complemented either by *S. meliloti fadD* or *E. coli fadD*, but constitutive expression of *sma0150* or *smb20650* could not complement for growth on oleate (Figure 1A). In order to be able to express pET vectors using the NOVAGEN expression system, we have created a *fadD* mutant of strain *E. coli* BL21(DE3). BfadD1 could not be complemented for growth on oleate by vectors expressing either *sma0150* or *smb20650*, while it could be complemented by *E. coli fadD* or *S. meliloti fadD* (Figure 1B). These data suggest that SMa0150 or SMb20650 do not have the capacity to form oleoyl-CoA that would support the growth of *E. coli* or *S. meliloti* cells deficient in long-chain acyl-CoA synthetase.

### 3.2. Free Fatty Acid Accumulation in Sinorhizobium meliloti and Escherichia coli fadD Mutants Cannot be Reverted by sma0150 or smb20650

**Figure 2:**
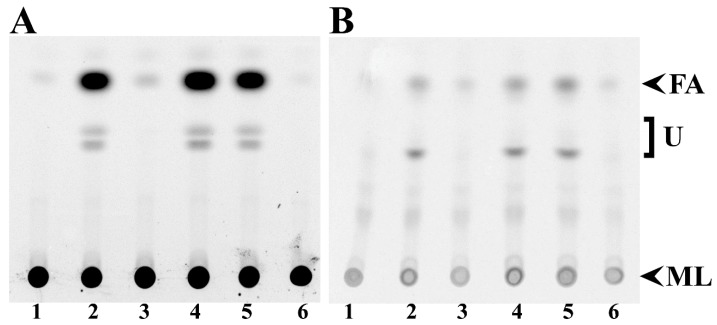
Expression of *sma0150* or *smb20650* does not abolish free fatty acid accumulation in *fadD* mutants of *S. meliloti*. Thin-Layer Chromatography (TLC) analyses of cellular lipid extracts (**A**) and lipid extracts of spent media (**B**) obtained either from wild type *S. meliloti* GR4 carrying the empty vector pNG28 (lane 1) or from its *fadD* mutant QS77 carrying either pNG28 (lane 2), pRCanul2 (*smc02162*, lane 3), pRCanul1 (*sma0150*, lane 4), pRCanul3 (*smb20650*, lane 5), or pRCanul 4 (*fadD_Ecoli_*, lane 6) grown on Robertsen MM into the stationary phase (OD_620_= 1.2, 66 h of growth). The membrane lipids (ML) did not migrate from the origin and the spot for fatty acids (FA) is indicated. U: unidentified lipid spots. A single experiment representative of three repetitions is shown.

We found that mutants in *fadD* of *S. meliloti* and *E. coli* accumulated free fatty acids in the stationary phase [3]. Such fatty acid accumulation does not occur in QS77 carrying the *fadD*-bearing plasmid pBBRD4 [3]. Thin-layer chromatography (TLC) analyses of lipid extracts from cells demonstrate that wild type *S. meliloti* GR4 cells carrying the empty vector pNG28 do not accumulate free fatty acids while its *fadD* mutant QS77 carrying pNG28 shows fatty acid accumulation (Figure 2A, lanes 1 and 2). The expression of *smc02162* (*fadD*) from pRCanul2 or of *E. coli fadD* from pRCanul4 suppresses fatty acid accumulation in the *fadD* mutant (Figure 2A, lanes 3 and 6). However, the expression of the two closest *S. meliloti fadD* homologs *sma0150* or *smb20650* into the *fadD* mutant QS77 does not suppress the fatty acid accumulation phenotype (Figure 2A, lanes 4 and 5). Similar effects were observed in culture supernatants although only a modest accumulation of fatty acids occurred (Figure 2B). The quantification of spots showed that labelled free fatty acids accumulated in supernatants were between 10% and 16% of the amount of free fatty acids accumulated in cells (Table S2 and Figure S3). Lipid extracts of cells and spent mediaof *S. meliloti* strains lacking *smc02162* or *E. coli fadD* accumulated other unidentified hydrophobic compounds in addition to free fatty acids (Figure 2). 

**Figure 3:**
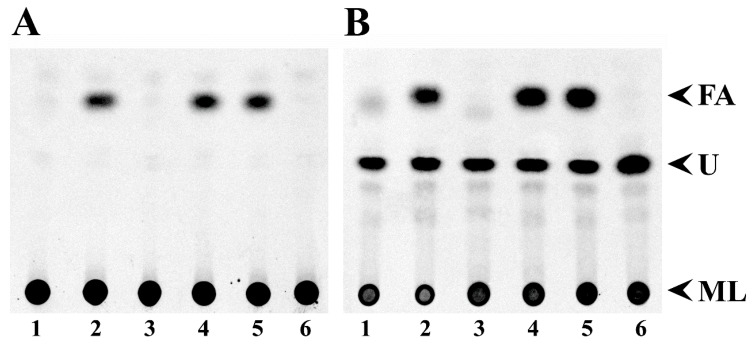
Expression of *sma0150* or *smb20650* does not abolish free fatty acid accumulation in *fadD* mutants of *E. coli*. TLC analyses of cellular lipid extracts (**A**) and lipid extracts of spent media (**B**) obtained either from wild type *E. coli* BL21 (DE3) pLysS carrying the empty vector pET17b (lane 1) or from its *fadD* mutant BfadD1 pLysS carrying pET17b (lane 2), pECH8 (*fadD_Ecoli_*, lane 3), pECH1 (*sma0150*, lane 4), pECH7 (*smb20650*, lane 5), or pECH6 (*smc02162*, lane 6), grown on M9 MM for 26 h. The membrane lipids (ML) did not migrate from the origin, and the spot for fatty acids (FA) is indicated. U: unidentified lipid spot. A single experiment representative of three repetitions is shown.

In a previous work, we have shown that a *fadD* mutant of *E. coli* strain Y-Mel accumulated free fatty acids. As for the case of strain Y-Mel [3], a *fadD* mutant of *E. coli* BL21(DE3) was accumulating significant amounts of free fatty acids (Figure 3A, lanes 2). The expression of *E. coli fadD* or *S. meliloti fadD* (*smc02162*) eliminated fatty acid accumulation (Figure 3A, lanes 3 and 6) but the expression of *sma0150* or *smb20650* in the *E. coli* mutant background did not eliminate free fatty acid accumulation (Figure 3A, lanes 4 and 5). In the spent media of *E. coli* BfadD1 derivatives that lack functional long-chain fatty acid-CoA ligase, a higher amount of free fatty acids to those found in cell extracts is observed (Figure 3B, Figure S4 and Table S3). The amount of labelled free fatty acids associated with cells were about 50% of the free fatty acids observed in supernatants (Table S3 and Figure S4). The bigger proportion of free fatty acids in the supernatants of *E. coli fadD* mutants might be due to the fact that *E. coli* cultures were in the stationary phase for more generation times than *S. meliloti* cultures. In the conditions studied, the generation time for *E. coli* was 2 h and for *S. meliloti* it was 8 h. Interestingly, the supernatants of all *E. coli* cultures present a hydrophobic compound with an R_f_ value similar to that of the unidentified compound observed in supernatants of *S. meliloti* lacking a functional *fadD* (Figure 2B, lanes 2, 4 and 5). However, in *E. coli* its presence is not correlated with the absence of FadD activity (Figure 3B).

### 3.3. Effect of the E. coli fadD, S. meliloti fadD (smc02162), and S. meliloti Genes sma0150 and smb20650 on the Surface Motility of a fadD Mutant of S. meliloti (QS77)

**Figure 4:**
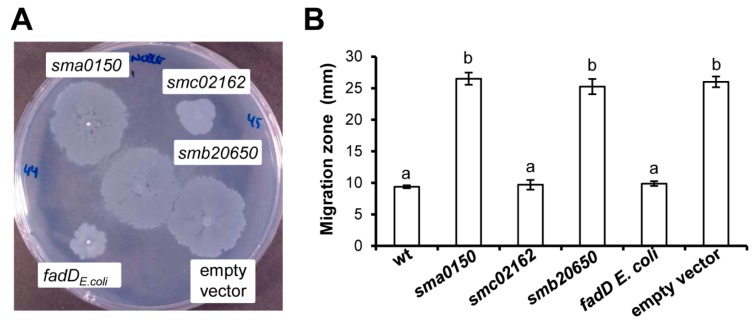
Effect of different *fadD* homologues on the surface motility of *S. meliloti* QS77 (*smc02162^−^*). (**A**) A representative picture of surface motility on Robertsen semisolid MM (0.6% Noble agar) shown by QS77 harbouring either pRCanul1 (*sma0150*), pRCanul2 (*smc02162*), pRCanul3 (*smb20650*), pNG28 (empty vector), or pRCanul 4 (*fadD_Ecoli_*). (**B**) The surface expansion shown by the wild type strain and QS77 derivatives shown in (**A**). The bars and error bars represent the mean and standard error of the migration zones obtained for each strain from two independent biological experiments with at least four technical replicates. Different letters indicate significant differences according to an analysis-of-variance test (*p* ≤ 0.05).

It has been shown that loss of function of *smc02162* (*fadD*) promotes surface motility in *S. meliloti* [2,32]. This phenotype is reverted to the wild type behaviour by introducing *smc02162* in trans in a pBBR1MCS-3 derivative construct [2]. To test if this effect is exerted exclusively by the *S. meliloti fadD* gene or by any other fatty acyl-CoA ligase, the motility behaviours of *fadD* mutant (QS77) derivatives expressing the *E. coli fadD* gene, or either genes coding for the two closest *S. meliloti* FadD homologues, *sma0150 (matB)* and *smb20650*, were assayed on semisolid Robertsen MM. As shown in Figure 4, only plasmids pRCanul2 and pRCanul4, containing *smc02162* and *E. coli fadD,* respectively, were able to inhibit surface translocation of the mutant to levels similar to those exhibited by the wild type strain (Figure 4B and Figure S5). These results indicate that the regulation of surface motility is specific to the long-chain fatty acyl-CoA ligase FadD.

### 3.4. Absence of fadD Reduces Survival Rates in the Stationary Phase of Growth

**Figure 5:**
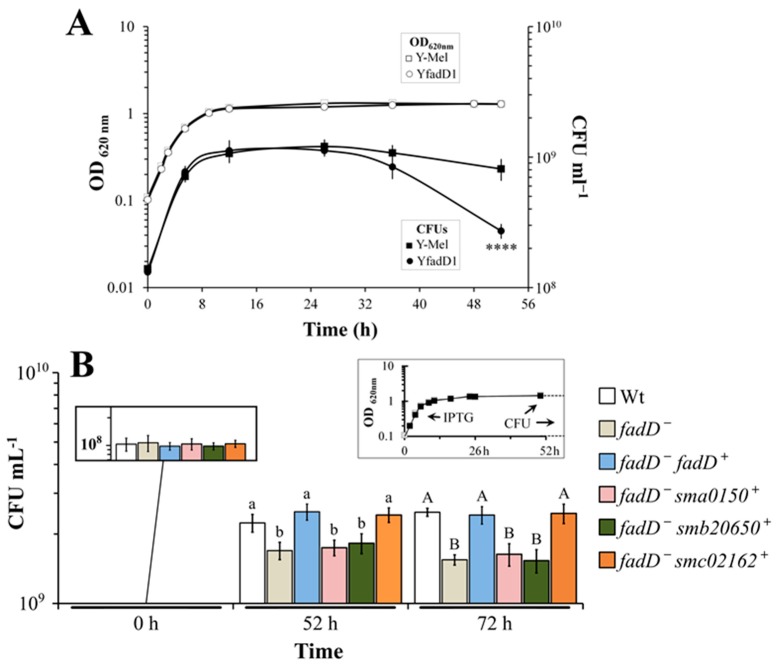
Absence of *fadD* reduces survival in the stationary phase of *E. coli*. (**A**) The growth curves and number of viable cells (CFU) of *E. coli* Y-Mel and its *fadD* mutant YfadD1 grown in M9 MM. Open symbols represent optical density (OD) whereas CFU ml^−1^ are represented with filled symbols. The statistical significance was calculated in Prism 8.4 using an unpaired two-tailed *t*-test in which the *fadD* mutant was compared to the parental strain. The statistical significance is shown (**** *p* <0.0001). (**B**) The number of viable cells of *E. coli* wild type BL21 (DE3) pLysS carrying the empty vector pET17b (Wt) or from its *fadD* mutant BfadD1 pLysS carrying pET17b (*fadD^−^*), pECH8 (*fadD^+^*), pECH1 (*sma0150*), pECH7 (*smb20650*), or pECH6 (*smc02162*) after 0, 52, and 72 h of growth in M9 MM. The inset in the upper right corner represents the OD of the cultures, and the arrows point to the addition of IPTG and to 52 h when CFU were determined. From 52 h to 72 h the OD of the different cultures maintained constant. For each strain three independent cultures were analysed. The error bars represent the SD. Different letters for bars at 52 and 72 h indicate significant differences according to an analysis-of-variance test (*p* < 0.001).

Since strains lacking a functional *fadD* cannot reutilize free fatty acids released from membranes as a carbon source, we speculated that the wild type should have a metabolic advantage over the mutant in the stationary phase [3]. In a first experiment with the strains Y-Mel and its *fadD* mutant YfadD, viable counts of the mutant strain after 52 h of growth were reduced to 33% with respect to the wild type strain (Figure 5A). Next, we checked for survival rates of strain BL21 (DE3) pLysS and its *fadD* mutant BfadD1 carrying either an empty plasmid or the expression plasmids for *E. coli fadD*, *S. meliloti fadD* (*smc02162*), or *S. meliloti genes sma0150* or *smb20650*. Although, to a lesser extent, again, a significant difference in colony forming units was observed between the wild type and its *fadD* mutant with a reduction of the survival to 75% at 52 h and to 62% at 72 h. Viable counts were similar to the wild type in strains complemented either with *E. coli* or *S. meliloti fadD*, while survival rates could not be restored by *sma0150* or *smb20650* (Figure 5B), probably due to the inability to consume free fatty acids.

**Figure 6:**
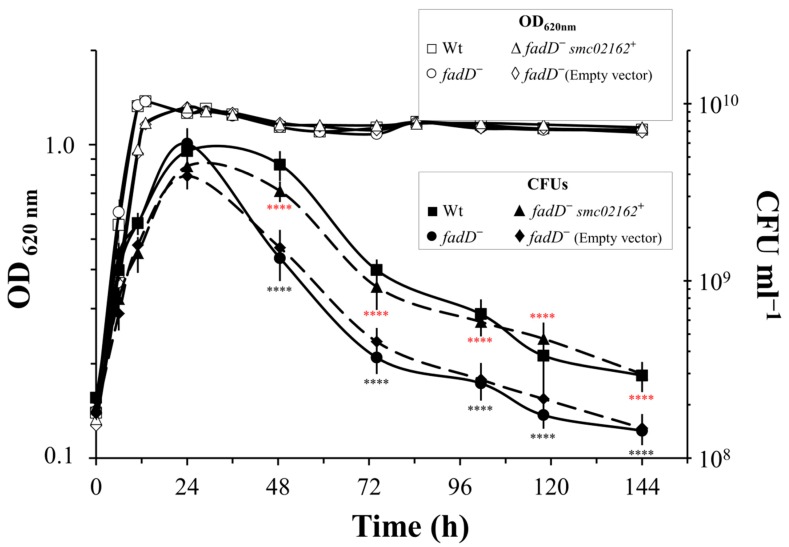
Expression of *fadD* in a *S. meliloti fadD* mutant recovers survival rate. The growth curves and viable cells (CFU) of wild type *S. meliloti* GR4, its *fadD* mutant QS77, QS77 harbouring pRCanul2 (*smc02162*), and QS77 harbouring empty plasmid pNG28 grown on Sherwood MM. Open symbols represent optical density, whereas CFU are represented with filled symbols. The statistical significance was calculated in Prism 8.4 using an unpaired two-tailed *t*-test in which the *fadD* mutant QS77 was compared to the parental strain GR4 (black asterisks), and the QS77 harbouring an empty plasmid was compared to the QS77 harbouring *smc02162* (red asterisks). The statistical significance is shown (**** *p* <0.0001). For each strain, three independent cultures were analysed. The error bars represent the SD.

In order to test if the absence of *fadD* also reduces viability in *S. meliloti*, we followed the optical density (OD) and the number of viable cells of cultures of *S. meliloti* GR4 and its *fadD* mutant QS77 in a long-term cultivation experiment (see material and methods). Even after 22 days of growth, the OD of the cultures remained constant and there was no difference between wild type and mutant (Figure S6). However, the number of viable cells of both cultures started to decrease after 7 days, and, after 9 days, a significant difference was observed between them. From day 9 to day 22, the number of colony forming units (CFUs) obtained from mutant cultures was decreased from 45% (day 14) to 30% (day 22) compared to those obtained from the wild type (Figure S6). When Sherwood MM [18] was used instead of Robertsen MM [17], a significant decrease in viability was already observed after 2 days of growth (Figure 6). This difference in survival might be due to the significantly lower concentration of carbon source present in Sherwood MM of 8.3 mM succinate versus 55 mM of mannitol present in Robertsen MM. The viability of wild type and *fadD* mutant was decreasing and the numbers of viable cells recuperated from the *fadD* mutant were less than 50% of those recovered from the wild type (Figure 6). Importantly, the *fadD* mutant carrying a plasmid harboring *fadD* shows a survival rate similar to the wild type, while the mutant with the empty vector is surviving to a lesser extent (Figure 6). Therefore, the presence of FadD confers an increased survival to cultures of *E. coli* and *S. meliloti* in the stationary phase.

## 4. Discussion

A mutant in the *fadD* of *S. meliloti* is unable to grow on oleate as the sole carbon source, shows increased surface motility with respect to the wild type, and accumulates free fatty acids in the stationary phase [2,3]. In this work, we have shown that all of these different phenotypes can be complemented by the expression of the gene encoding the *E. coli* FadD homologue, indicating that there are no functional differences between the *S. meliloti* and the *E. coli* FadD proteins. However, none of these phenotypes could be reverted by any of the closest *S. meliloti* FadD homologues, SMa0150 or SMb20650. Based on our in vivo experiments, we can conclude that neither SMa0150 nor SMb20650 have the capacity of linking long-chain fatty acids efficiently to CoA.

The *fadD* mutants of *S. meliloti* accumulate free fatty acids at the entry of the stationary phase and the source for them are mainly membrane phospholipids [3]. However, little is known about the activities that release fatty acids. Recently, we have identified in *S. meliloti* a diacylglycerol lipase that is, in part, responsible for the release of fatty acids [33]. In the absence of FadD, free fatty acids are accumulated in the stationary phase, while in wild type strains the fatty acids are consumed by β-oxidation [3]. We hypothesized that this extra carbon source available for the wild type might confer a survival advantage in comparison with their counterparts that lack FadD activity and are therefore unable to utilize the accumulated fatty acids. Indeed, inactivation of *fadD* in *E. coli* Y-Mel reduces its survival growth to 33% with respect to the wild type strain (Figure 5A). In similar conditions of growth, a *fadD* mutant of *E. coli* BL21(DE3) lost 25% of its viability with respect to the wild type (Figure 5B). Importantly, cell viability is restored to wild type levels when such a mutant is complemented with *S. meliloti* or *E. coli fadD* but not after the expression of *sma0150* or *smb20650* (Figure 5B). Different investigations have made use of *E. coli fadD* mutants. Fulda et al. [34] cloned and sequenced the *E. coli* K12 *fadD* gene by complementing the *fadD* phenotype with different deletion clones. Moreover, an *E. coli* BL21(DE3) derivative mutated in *fadD* was used to test for complementation of growth on oleate with five different long-chain acyl-CoA synthetases from rats. Only one of them could complement for growth on oleate [35]. Complementation of an *E. coli fadD* mutant by the expression of *fadD* from *E. coli* restored its ability to grow on C12, as well as growth on the non-inducing fatty acids of β-oxidation C10 and C8. These results show that FadD dosage plays an important role in the regulation of β-oxidation [36]. 

Most bacteria reduce their size considerably upon entry into the stationary phase as a result of reductive division and dwarfing. Degradation of the membrane components is part of the dwarfing process of non-differentiating bacteria under starvation for exogenous carbon and energy generating small, coccoid cells (reviewed in [37]). Farewell et al. [38] found in *E. coli* an increased expression of genes of the FadR regulon during the entry of cells into the stationary phase and mutants unable to increase their expression survive long-term stasis poorly. These authors suggested that the Fad regulon, apart from being required for growth on exogenous long-chain fatty acids, might be involved in providing the growth arrested cells with endogenous carbon and energy during dwarfing [38]. Comparing the cells of *S. meliloti* Rm1021 at the entry of the stationary phase with cells in the exponential phase of growth, Sauviac et al. [39] found a significant up-regulation of the operon *smc02229-fadAB* that is required for fatty acid β-oxidation. The M values of this microarray study for the comparison of stationary phase versus exponential phase of growth for the genes *smc02229* (*fadE*), *fadA*, and *fadB* were 3, 2.7, and 2.8, respectively [40]. We compared the global expression of cultures of *S. meliloti* Rm1021 at the entry of the stationary phase for the wild type strain and its *fadD* mutant and found strong up-regulation of fatty acid degradation genes in the wild type strain [3]. The *fadD* mutant accumulates about 100 nmol of free fatty acids associated to the cells per ml of culture, while the wild type contains less than 1 nmol/mL culture. Given the strong induction of fatty acid degradation genes, we suggested that free fatty acids were consumed in the wild type and accumulated in the *fadD* mutant. Furthermore, the *fadD* mutants of *E. coli* accumulated a significant amount of free fatty acids both in the cell-associated fraction and in the culture supernatant ([3] and Figure 3). We propose that the higher survival in the stationary phase observed for the wild type strains of *S. meliloti* and of *E. coli* with respect to their *fadD* mutants (Figure 5 and Figure 6) is due to the capacity of the wild type to metabolize endogenous fatty acids. 

The β-oxidation of fatty acids has usually been studied as a property to utilize exogenous long-chain fatty acids [1]. However, our present and previous results emphasize the important role of the fatty acid degradation system in the utilization of endogenous fatty acids. The capacity to use endogenous fatty acids provides the cells with extra carbon source during starvation, which results in a better rate of survival in the stationary phase. In *E. coli*, an alternative complete β-oxidation system has been described that works under anaerobic conditions in the presence of nitrate or fumarate as terminal electron acceptors [41]. ORFs coding for this pathway are found in the genomes of *E. coli*, *Salmonella*, *Klebsiella*, *Yersinia*, and *Vibrio*. It is likely that the anaerobic β-oxidation system gives them an important extra carbon source under starving conditions.

The biochemical function of long-chain fatty acyl-CoA synthetase is necessary for activation of fatty acids, thereby preparing them for subsequent degradation by β-oxidation. However, this enzyme activity also affects a number of different phenotypes. Several studies suggest a role of long-chain fatty acyl-CoA synthetase in pathogenesis since the inactivation of *fadD* affects virulence or colonization in *Pseudomonas aeruginosa* [13], *Salmonella enterica*, serovar Typhimurium [42], and *Neisseria meningitidis* [43]. The expression of *fadD* is involved in antibiotic production in *Streptomyces coelicolor* [44]. Furthermore, a lack of long-chain fatty acyl-CoA synthetase in the fungal pathogen *Candida albicans* led to a significant reduction in metabolic activity during biofilm formation [45], while the β-oxidation of fatty acids is required for fruiting body development in *Myxococcus xanthus* [46].

## 5. Conclusions

The capacity of fatty acid utilization from the extracellular environment has been described for many Gram-negative as well as for Gram-positive bacteria [43,47] and for yeast [45,48]. It is likely that all microorganisms, pathogenic or environmental, with the capacity to degrade fatty acids benefit from the utilization of exogenous as well as endogenous fatty acids. Our data reinforce the importance of a functional system for fatty acid degradation in providing a better survival rate in the stationary phase.

## Figures and Tables

**Table 1:** Bacterial strains and plasmids used in this work.

Strain or Plasmid	Relevant Characteristics^a^	Reference or Source
*Escherichia coli*		
DH5α	*recA1,* Φ80 *lacZ∆M1*; cloning strain	[21]
S17-1	*thi pro recA hsdR^−^ hsdM*^+^ RP4 integrated in the chromosome, 2-Tc::Mu, Km::Tn*7* (Tp^R^/Sm^R^)	[22]
Y-Mel	Wild type strain	[23]
YfadD1	Y-Mel *fadD::kan*	[3]
JW1794-1	BW25113 *fadD::kan*	[19]
BL21(DE3)	F^−^*ompT hsdSB* (rB^−^, mB^−^) *gal dcm* (DE3)	[24]
BfadD1	BL21(DE3) *fadD::kan*	This work
*Sinorhizobium meliloti*		
GR4	Wild type strain	[25]
QS77	*fadD::*Tn*5* insertion mutant derivative of GR4, Nm^R^	[2]
Plasmids		
pLysS	Cm^R^; causes repression of T7 polymerase	[24]
pET16b	Expression vector, Cb^R^	Novagen
pET17b	Expression vector, Cb^R^	Novagen
pAL55	*smb20650* in pBBR1MCS-5, Gm^R^	[5]
pECH1	*sma0150* in pET16b, Cb^R^	This work
pECH6	*smc02162* in pET17b, Cb^R^	This work
pECH7	*smb20650* in pET17b, Cb^R^	This work
pECH8	*E. coli fadD* pET16b, Cb^R^	This work
pBBR1MCS-3	Broad-host range vector, Tc^R^	[26]
pBBRD4	pBBR1MCS-3 derivative harbouring the *fadD* gene of *S. meliloti* GR4, Tc^R^	[2]
pRK404	Broad-host range vector, Tc^R^	[27]
pRCanul1	pECH1 cloned as a *Bam*HI fragment into pRK404, *sma0150*	This work
pRCanul2	pECH6 cloned as a *Hind*III fragment into pRK404, *smc02162*	This work
pRCanul3	pECH7 cloned as a *Bgl*II fragment into pRK404, *smb20650*	This work
pRCanul4	pECH8 cloned as a *Bam*HI fragment into pRK404, *fadD_Ecoli_*	This work
pNG28	pET17b cloned in pRK404	[28]

**^a^**Tc^R^, Tp^R^, Sm**^R^**, Km^R^, Nm^R^, Cm^R^, Gm^R^, Cb^R^: tetracycline, trimethoprim, streptomycin, kanamycin, neomycin, cloramphenicol, gentamicin, and carbenicillin resistance, respectively.

## Supplementary Materials

The following are available online at https://www.mdpi.com/2076-2607/8/4/470/s1: Figure S1: Multiple sequence alignments of *S. meliloti* Rm1021 FadD (SMc02162) with *E. coli* FadD (FadDEc) and different ORFs of *S. meliloti* Rm1021 with homology to SMc02162; Figure S2: Multiple sequence alignments of SMa0150 with characterized malonyl-CoA synthetases; Figure S3: Graphical representation of spot intensities of free fatty acids formed in different *S. meliloti* strains; Figure S4: Graphical representation of spot intensities of free fatty acids formed in different *E. coli* strains; Figure S5: Effect of different *fadD* homologues on the surface motility of *S. meliloti* QS77 (*smc02162^−^*); Figure S6: Absence of *fadD* reduces survival in the stationary phase of *S. meliloti*; Table S1: Results of the pairwise sequence alignments of SMc02162 (*S. meliloti* FadD) with *E. coli* FadD or with different acyl-CoA synthetases of the *S. meliloti* Rm1021 genome; Table S2: Quantification of free fatty acids in different *S. meliloti* strains; Table S3: Quantification of free fatty acids in different *E. coli* strains.
