# Supplementary material for: Role of Sinorhizobium meliloti and Escherichia coli Long-Chain Acyl-CoA Synthetase FadD in Long-Term Survival
Source: Microorganisms. 2020 Mar 26;8(4):470. doi: 10.3390/microorganisms8040470 (PMC7232532; doi:10.3390/microorganisms8040470)
Supplement: Supplementary file 1 [file microorganisms-08-00470-s001.pdf]

microorganisms

MDPI

Supplementary Materials

Table S1. Results of pairwise sequence alignments of SMc02162 (*S. meliloti* FadD) with *E. coli* FadD or with different acyl-CoA synthetases of the *S. meliloti* Rm1021 genome[a].

Locus_tag (ID)	Query coverage	Score	% identity	% similarity	E-value	Annotation
FadD *E. coli*	15–565	557.9	55.7	81.2	3.5e-163	long-chain fatty acid-CoA ligase FadD
SMa0150 (MatB)	54–565	200.2	31.1	59.2	1.6e-65	malonyl-CoA synthetase
SMb20650	39–562	190.7	29.6	57.1	1.1e-52	long-chain fatty acid-CoA ligase
SMc00261	29–565	182.4	27.5	56.1	3.8e-50	Putative fatty acid-CoA ligase
SMc00741 (AcsA3)	60–565	133.9	27.4	52.2	1.6e-35	Putative acetyl-CoA synthetase
SMc04095	61–565	108.7	23.8	53.5	6.7e-28	Putative acyl-CoA synthetase
SMc04093 (AcsA1)	50–565	103.1	21.5	51.0	3.4e-26	Probable acetyl-coenzyme A synthetase
SMc00965	52–565	87.1	23.7	51.5	1.7e-21	Putative acyl-CoA synthase
SMc00592	213–565	78.5	25.8	54.6	1.2e-18	Hypothetical, transmembrane protein
SMc00774 (AcsA2)	61–565	49.6	21.0	50.1	4.5e-10	Acetoacetyl-coenzyme A synthetase

[a]*S. meliloti* Rm1021 protein sequences and their annotation were obtained from https://iant.toulouse.inra.fr/bacteria/annotation/cgi/rhime.cgi (accessed on March 18, 2020). Data of pairwise alignment of SMc02162 with the different ORFs were obtained at the server https://www.ebi.ac.uk/Tools/psa/lalign/ (accessed on March 18, 2020).

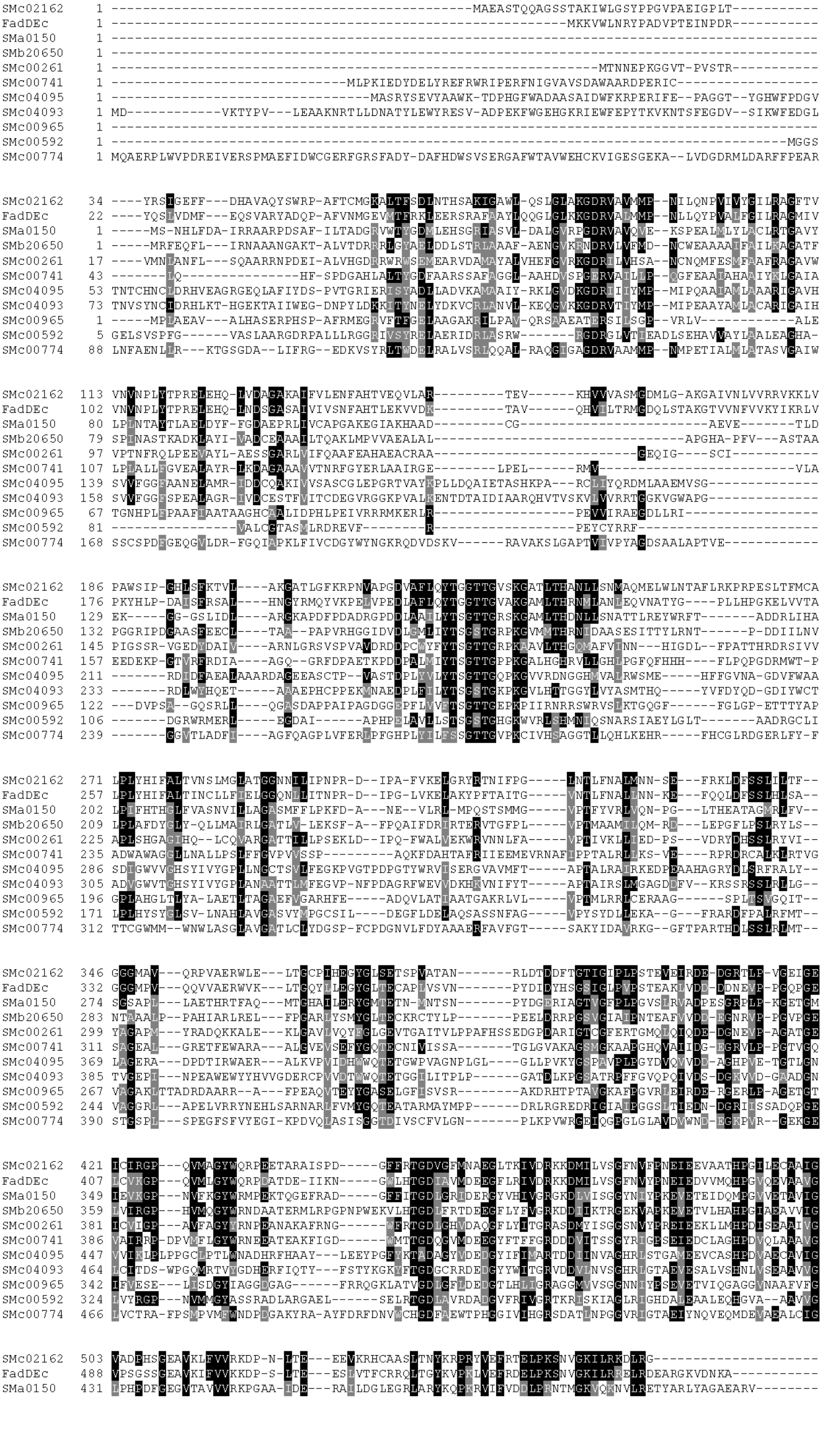

```
SMb20650  446 VPDPVLGAAIGALVVLSDP-T-LTE---KDIIRHCSRHLEDEMVPKIVEFRTELPKTDTGKVSRRLAAETLEPAE--------------
SMc00261  462 VPDPVWGEVGIAVCVARGGAT-VGA---AALREWLDGKIARYKLPKKIVFWSEMPKSAYGKITKKLIREELERRGELDIGSAGIGERR--
SMc00741  469 KPDPLRTEIVKAYVVLKPGVA-AGDETAAGIRDWVKNRLSMHEYPREIAFVDSLPLTTSGKVIRRLLREKAAAEARAASGG---------
SMc04095  534 IADPLKGQVPAGFLVINANVSRETEEIEKEVVGLVRERIGPVAAFRTAVCVKRLPKTRSGKILRSTIQKIIDRQPWT--MPATIDDPAIL
SMc04093  550 YPHPIKGQGIYCYVSLMAGEV-GDDELRQALVKHVRSEIGPIATPDKIQFAPGLPKTRSGKIMRRILRKIAEDDFGSLGDTSTLADPGVV
SMc00965  423 LHHPDLGSELAAVIEPGDG---FDH---AALERHLAADLPRYKRPRRIWLCRKMPMTASGKVAAGELRQWIATENSALERLV--------
SMc00592  403 NDTE----IHAYYVGSGDP---------DEVRRLLVEASGLTLMHVGASRLDKLPRLTSGKIDYPALRMRPAATCG------SSEEGDV
SMc00774  554 QDWEDDVR-VVLFVRLARGVE-LTEALTREIKNRIRSGASPRHVPAKIIAVADIPRTKSGKIVELAVRDVVHGRPVKNKE--ALANPEAL

SMc02162      -----------------------------------------------------------------
FadDEc        -----------------------------------------------------------------
SMa0150       -----------------------------------------------------------------
SMb20650      -----------------------------------------------------------------
SMc00261  546 -------------------------------------------------------SAAP------
SMc00741      -----------------------------------------------------------------
SMc04095  622 DEITELLR-----------------------------------------------SKGIGV----
SMc04093  639 DDLIANRQ-----------------------------------------------NRA-------
SMc00965      -----------------------------------------------------------------
SMc00592  474 EALFAQLFYPKKVRPEDSFLSLGGDSLRFVQLSIGLEKRLGELPEGWEKMPVGALAAHNGRERKSRRIGADFLI
SMc00774  640 DL-FAGLEELKS-----------------------------------------------------
```

Figure S1. Multiple sequence alignments of *S. meliloti* Rm1021 FadD (SMc02162) with *E. coli* FadD (FadDEc) and different ORFs of *S. meliloti* Rm1021 with homology to SMc02162 (https://iant.toulouse.inra.fr/bacteria/annotation/cgi/rhime.cgi, accessed on March 18 th, 2020). Details of the different ORFs are given on Table S1. Alignments were performed using Clustal Omega at EMBL-EBI (https://www.ebi.ac.uk/Tools/msa/clustalo/, accessed on March 18th, 2020) [1] and the figure was created at the BoxShade server using the option of consensus to SMc02162 sequence (https://embnet.vital-it.ch/software/BOX_form.html, accessed on March 18th, 2020). SMc00592 is a multidomain protein with a total of 885 aminoacids. Only the first 547 amino acids have been used in the alignment. Amino acids identical to those in SMc02162 are shaded in black while amino acids similar to those present in SMc02162 are shaded in grey.

```
SMa0150     1 --------------------------------------MSNHLFDAIRR---AARPDSAFILTADGRVWT
Rl MatB     1 --------------------------------------MSNHLFDAMRA---AAPGNAPFIRIDNTRTWT
Bd MatB     1 ------------------------------MNRAANANLFSRLFDGLD------DPKRLAIETHDGARIS
Sc MatB     1 ---------------------------------------MSSLFPALSPAPTGAPADRPA-LRFGERSLT
At AAE3     1 ---------------------------------------MEVFKAAFSEA--SNSCDRIA-IKADGKSYS
Hs ACSF3    1 MPPHVVLTFRRLGCALASCRLAPARHRGSGLLHTAPVARSDRSAPVFTRA--LAFGDRIA-LVDQHGRHT

SMa0150    30 YGDMLEHSG---------------RIASVLDALGVRPGDRVAVQVEKSPEALMLYLACLRTGAVYLPLNT
Rl MatB    30 YDDAFALSG---------------RIASAMDALGIRPGDRVAVQVEKSAEALILYLACLRSGAVYLPLNT
Bd MatB    35 YGDLIARAG---------------QMANVLVARGVKPGDRVAVQVEKSVANIVLYLATVRAGAVYLPLNT
Sc MatB    31 YAELAAA---------------AGATAGRIGG-----AGRVAVWATPAMETGVAVVAALLAGVAAVPLNP
At AAE3    29 YGQLTSSALRISKLFLKDDTTNGGQETKKYEGFGSLKGARIGIVAKPSAEFVAGVLGTWFSGGVAVPLAL
Hs ACSF3   68 YRELYSRSLRLSQEICRLCGCVG----------GDLREERVSFLCANDASYVVAQWASWMSGGVAVPLYR

SMa0150    85 AYTLAELDYFFGDAEPRLIVCAPGAKEGIAKHAADCGAEVETLDEKGGGSLIDLARG--KAPDFPDADRG
Rl MatB    85 AYTLAELDYFIGDAEPRLVVVASSARAGVETIAKPRGAIVETLDAAGSGSLLDLARD--EPADFVDASRS
Bd MatB    90 AYTLNELDYFIGDAEPSLVVCDPSKAEGLAPIAAKVKAGVETLGPDGKGSLTEAADK--ASSAFTTVPRE
Sc MatB    81 KSGDKELAHILSDSAPSLVLAPPDAE----------------LPPALGA-LERVDVDVRARGAVPEDGAD
At AAE3    99 SYPEAELLHVMNDSDISLLLSTEDHSETMKTIAAKSGARFHLIPPVVNSTSETVACNQFQDDSFEAEGKF
Hs ACSF3  128 KHPAAQLEYVICDSQSSVVLVSQEYLELLSPVVRKLGVPLLPLTPAIYTG----AVEEPAEVPVPE-QGW

SMa0150   153 PDDLAAILYTSGTTGRSKGAMLTHDNLLSNATTLREYWRFTADDRLIHALPIFHTHGLFVASNVILLAGA
Rl MatB   153 ADDLAAILYTSGTTGRSKGAMLTHGNLLSNALTLRDFWRVTAGDRLIHALPIFHTHGLFVATNVTLLAGA
Bd MatB   158 NDDLAAILYTSGTTGRSKGAMLTHDNLASNSLSLVGYWRFTDKDVLIHALPIYHTHGLFVATNVTLFSRA
Sc MatB   134 DGDPALVVYTSGTTGPPKGAVIPRRALATTLDALADAWQWTGEDVLVQGLPLFHVHGLVLGILGPLRRGG
At AAE3   169 LDDPALIVYTSGTTGKPKGVVHTHNSINSQVRMLTEAWEYTSADHFLHCLPLHHVHGLFNALFAPLYARS
Hs ACSF3  193 RNKGAMIIYTSGTTGRPKGVLSTHQNIRAVVTGLVHKWAWTKDDVILHVLPLHHVHGVVNALLCPLWVGA

SMa0150   223 SMFFLPKFDANEVLR------------LMPQSTSMMGVPTFYVRLVQNPGL-----------THEATAGM
Rl MatB   223 SMFLLSKFDPEEILS------------LMPQATMLMGVPTFYVRLLQSPRL-----------DKQAVANI
Bd MatB   228 SMIFLPKLDPDLIIK------------LMARATVLMGVPTFYTRLLQNAAL-----------SRETTRHM
Sc MatB   204 SVRHLGRFSTEGAAR-----------ELNDGATMLFGVPTMYHRIAETLPADPE--------LAKALAGA
At AAE3   239 LVEFLPKFSVSGIWRRWRESYPVNDEKTNDSITVFTGVPTMYTRLIQGYEAMDKE---MQDSSAFAARKL
Hs ACSF3  263 TCVMMPEFSPQQVWEKFLSS-------ETPRINVFMAVPTIYTKLMEYYDRHFTQPHAQDFLRAVCEEKI

SMa0150   270 RLFVSGSAPLLAETHRTFAQMTGHAILERYGMTETNMNTSNPYDGE-RIAGTVGFPLPGVSLRVADPESG
Rl MatB   270 RLFISGSAPLLAETHTEFQARTGHAILERYGMTETNMNTSNPYEGK-RIAGTVGFPLPDVTVRVTDPATG
Bd MatB   275 RLFISGSAPLLAETHREWSARTGHAVLERYGMTETNMNTSNPYDGE-RVPGAVGFPLPGVSLRVTDPETG
Sc MatB   255 RLLVSGSAALPVHDHERIAAATGRRVIERYGMTETLMNTSVRADGE-PRAGTVGVPLPGVELRLVEEDGT
At AAE3   306 RLMMSGSSALPRPVMHQWESITGHRLLERYGMTEFVMAMSNPLRGA-RNAGTVGKPLPGVEAKIKEDEN-
Hs ACSF3  326 RLMVSGSAALPLPVLEKWKNITGHTLLERYGMTEIGMALSGPLTTAMRLPGSVGTPLPGVQVRIVSENPQ

SMa0150   339 -------------------RPLPKGETGMIEVKGPNVFKGYWRMPEKTQGEFRADGFFITGDLGRIDERG
Rl MatB   339 -------------------LALPPEQTGMIEIKGPNVFKGYWRMPEKTAAEFTADGFFISGDLGKIDRDG
Bd MatB   344 -------------------KELPREEIGMIEVKGPNVFKGYWRMPEKTKAEFRPDGFFITGDLGKIDGKG
Sc MatB   324 P-----------------IAALDGESVGEIQVRGPNLFTEYLNRPDATAAAFTEDGFFRTGDMAVRDPDG
At AAE3   374 ----------------------DANGVGEICVKSPSLFKEYWNLPEVTKESFTEDGYFKTGDAGRVDEDG
Hs ACSF3  396 REACSYTIHAEGDERGTKVTPGFEEKEGELLVRGPSVFREYWNKPEETKSAFTLDGWFKTGDTVVF-KDG
```

```
SMa0150   390 YVHIVGRG-KDLVISGGYNIYPKEVETEIDQMPGVVETAVIGLPHPDFGEGVTAVVVRKPGAA-------
Rl MatB   390 YVHIVGRG-KDLVISGGYNIYPKEVEGEIDQIEGVVESAVIGVPHPDFGEGVTAVVVRKPGAA-------
Bd MatB   395 YVHILGRG-KDLVISGGFNVYPKEIESEIDAMPGVVESAVIGVPHADFGEGVTAVLVCNKGAE-------
Sc MatB   377 YVRIVGRKATDLIKSGGYKIGAGEIENALLEHPEVREAAVTGEPDPDLGERIVAWIVPADPAAP------
At AAE3   422 YYVILGRNSADIMKVGGYKLSALEIESTLLEHPTVAECCVLGLTDNDYGEAVTAIIIAESAAKKRREDES
Hs ACSF3  465 QYWIRGRTSVDIIKTGGYKVSALEVEWHLLAHPSITDVAVIGVPDMTWGQRVTAVVTLREGHS-------


SMa0150   452 ---IDERAILDGLEGRLARYKQPKRVIFVDDLPRNTMGKVQKNVLRETYARLYAGAEARV
Rl MatB   452 ---LDEKAIVSALQDRLARYKQPKRIIFAEDLPRNTMGKVQKNILRQQYADLYTRT----
Bd MatB   457 ---VSEASVLKALDGRLAKFKMPKRVFVVDELPRNTMGKVQKNVLRDTYKDIYAKK----
Sc MatB   441 ---PALGTLADHVAARLAPHKRPRVVRYLDAVPRNDMGKIMKRALNRD------------
At AAE3   492 KPVITLEELCGWAKDKLAPYKLPTRLLIWESLPRNAMGKVNKKELKKSLENQE-------
Hs ACSF3  528 ---LSHRELKEWARNVLAPYAVPSELVLVEEIPRNQMGKIDKKALIRHFHPS--------
```

Figure S2. Multiple sequence alignments of SMa0150 with characterized malonyl-CoA synthetases from *Rhizobium leguminosarum* (Rl MatB), *Bradyrhizobium diazoefficiens* (Bd MatB), *Streptomyces coelicolor* (Sc MatB), *Arabidopsis thaliana* (At AAE13) and *Homo sapiens* (Hs ACSF3). Alignments were performed using Clustal Omega at EMBL-EBI (https://www.ebi.ac.uk/Tools/msa/clustalo/ accessed on February 1, 2020 [1]) and the figure was created at the BoxShade server (https://embnet.vital-it.ch/software/BOX_form.html accessed on 1st February 2020). The conserved motif ERYGMTE found in malonyl-CoA synthetases is shown with a line of red stars (*). Amino acids identical in at least three of the sequences are shaded in black and similar amino acids are shaded in grey.

Table S2. Quantification of free fatty acids in different *S. meliloti* strains. Spot intensities corresponding to free fatty acids observed on TLCs plate autoradiograms of *S. meliloti* ^{14}C-acetate-labeled cultures. Intensity is expressed as arbitrary units (x10^{6}). Average and SD are from three independent experiments.

S. meliloti strain	Cell associated	Spent media	Ratio cell associated/spent media
GR4 (pNG28)	2.57 ± 0. 42	2.39 ± 0.64	1.07
QS77 (pNG28)	94.1 ± 26.07	15.2 ± 8.81	6.19
QS77 (pRCanul2) *smc02162*	3.26 ± 1.60	2.74 ± 0.25	1.19
QS77 (pRCanul1) *sma0150*	118 ± 3.51	12.4 ± 4.62	9.52
QS77 (pRCanul3) *smb20650*	114 ± 3.0	13.2 ± 3.33	8.64
QS77 (pRCanul4) *fadD*$_{Ecoli}$	2.37 ± 0.09	2.41 ± 0.38	0.98

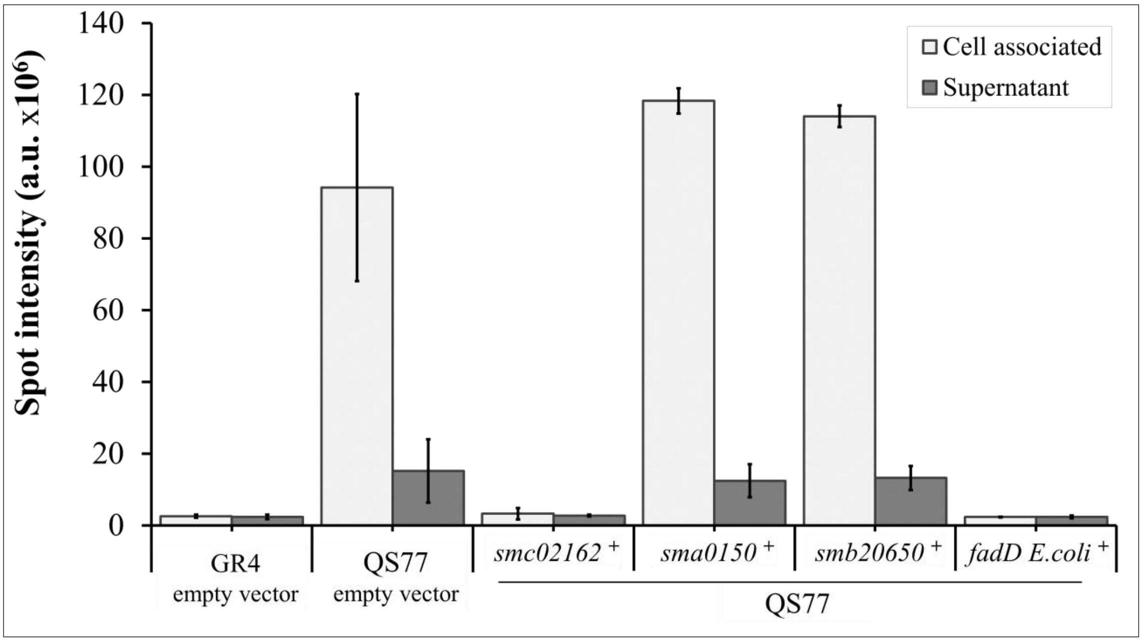

Figure S3: Graphical representation of spot intensities of free fatty acids formed in different *S. meliloti* strains. Bars correspond to spot intensities observed on TLCs plate autoradiograms in the indicated *S. meliloti* strains as shown in Table S1. Error bars represent SD calculated from three independent experiments.

Table S3: Quantification of free fatty acids in different *E. coli* strains. Spot intensities corresponding to free fatty acids observed on TLCs plate autoradiograms of *E. coli* ^{14}C-acetate-labeled cultures. Intensity is expressed as arbitrary units (x10^{5}). Average and SD are from three independent experiments.

E. coli strain	Cell associated	Spent media	Ratio cell associated/spent media
BL21(DE3) pLysS pET17b	3.43 ± 1.78	6.54 ± 2.20	0.52
BfadD1 pLysS pET17b	39.4 ± 10.96	59.4 ± 18.40	0.66
BfadD1 pLysS pECH8 *fadD$_{Ecoli}$*	2.78 ± 1.42	2.69 ± 1.16	1.03
BfadD1 pLysS pECH1 *sma0150*	38.1 ± 4.74	73.0 ± 32.33	0.52
BfadD1 pLysS pECH7 *smb20650*	33.0 ± 7.15	80.3 ± 28.56	0.41
BfadD1 pLysS pECH3 *smc02162*	3.47 ± 1.98	3.71 ± 2.20	0.93

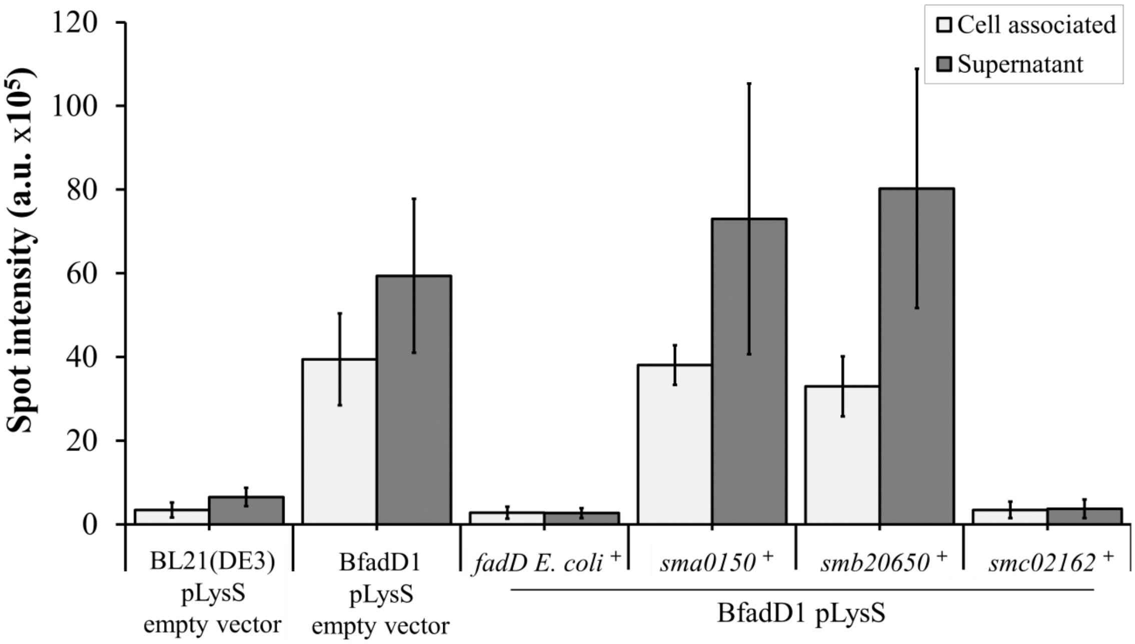

Figure S4: Graphical representation of spot intensities of free fatty acids formed in different *E. coli* strains. Bars correspond to spot intensities observed on TLCs plate autoradiograms in the indicated *E. coli* strains as shown in Table S2. Error bars represent SD calculated from three independent experiments.

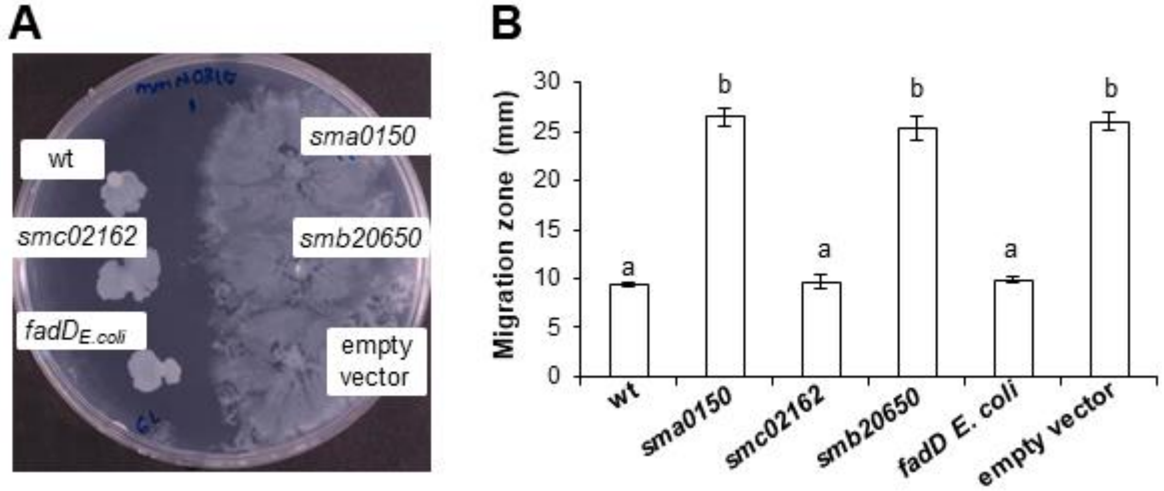

Figure S5. Effect of different *fadD* homologues on the surface motility of *S. meliloti* QS77 (*smc02162*$^-$). (**A**) Representative picture of surface motility on Robertsen semisolid MM (0.6% Noble agar) shown by the wild-type strain (wt) and QS77 harbouring either pRCanul1 (*sma0150*), pRCanul2 (*smc02162*), pRCanul3 (*smb20650*), pNG28 (empty vector), or pRCanul 4 (*fadD*$_{Ecoli}$). (**B**) Surface expansion shown by wt and QS77 derivatives shown in (**A**). Bars and error bars represent the mean and standard error of the migration zones obtained for each strain from two independent biological experiments with at least four technical replicates. Different letters indicate significant differences according to an analysis-of-variance test ($p \leq 0.05$).

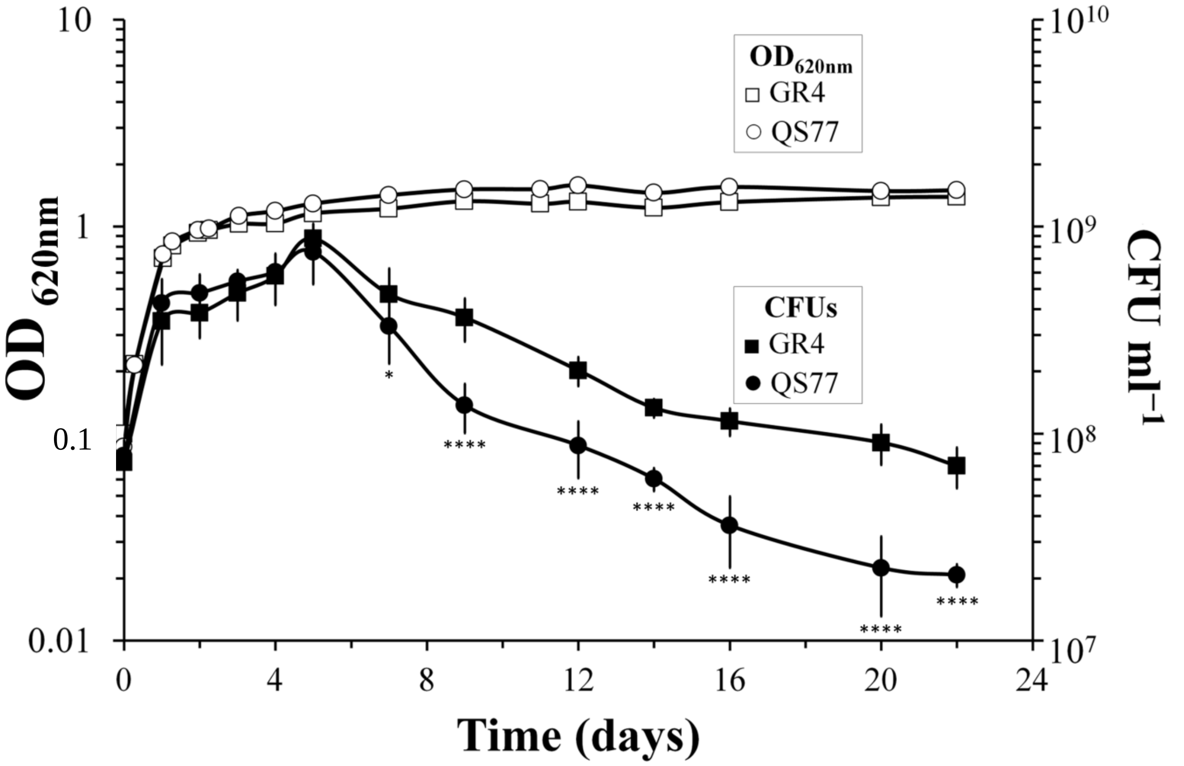

Figure S6. Absence of *fadD* reduces survival in stationary phase of *S. meliloti*. Growth curves and viable cells (CFU ml^{-1}) of *S. meliloti* GR4 and its *fadD* mutant QS77 grown on Robertsen MM. Open symbols represent optical density (OD) whereas CFU are represented with filled symbols. Statistical significance was calculated in Prism 8.4 using an unpaired two-tailed *t*-test in which the *fadD* mutant was compared to the parental strain. Statistical significance is shown (* $p < 0.05$; **** $p < 0.0001$). For each strain three independent cultures were analyzed. The error bars represent the SD.

Reference

1. Madeira, F.; Park, Y.M.; Lee, J.; Buso, N.; Gur, T.; Madhusoodanan, N., Basutkar, P.; Tivey, A.R.N.; Potter, S.C.; Finn, R.D.; et al. The EMBL-EBI search and sequence analysis tools APIs in 2019. *Nucleic Acids Res.* **2019**, *47*, W636–W641, doi:10.1093/nar/gkz268.
